# Fibrogenesis in Chronic DSS Colitis is Not Influenced by Neutralisation of Regulatory T Cells, of Major T Helper Cytokines or Absence of IL-13

**DOI:** 10.1038/s41598-019-46472-6

**Published:** 2019-07-11

**Authors:** Brecht Creyns, Jonathan Cremer, Tomoaki Hoshino, Karel Geboes, Gert de Hertogh, Marc Ferrante, Séverine Vermeire, Jan L. Ceuppens, Gert Van Assche, Christine Breynaert

**Affiliations:** 1 KU Leuven, Department of Microbiology, Immunology and Transplantation, Allergy and Clinical Immunology Research Group, Leuven, Belgium; 2 KU Leuven, Department of Chronic Diseases, Metabolism and Ageing, Translational Research Center for Gastrointestinal Disorders (TARGID), Leuven, Belgium; 3 Division of Respirology, Neurology and Rheumatology, Department of Internal Medicine, Kurume University School of Medicine, Kurume, Japan; 4 KU Leuven, Department of Imaging and Pathology, Translational Cell & Tissue Research, Leuven, Belgium; 5 University Hospitals Leuven, Department of Gastroenterology and Hepatology, Leuven, Belgium; 6 University Hospitals Leuven, Department of General Internal Medicine, Leuven, Belgium

**Keywords:** Inflammatory bowel disease, Crohn's disease

## Abstract

Mechanisms underlying fibrogenesis in chronic colitis are largely unknown. There is an urgent need for clinical markers and identification of targets to prevent, treat and limit intestinal fibrosis. This study investigated the contribution of major T cell cytokines and T regulatory cells (Tregs) to inflammation and fibrosis induced in a model of experimental colitis by oral intake of dextran sodium sulphate (DSS) in wild type and IL-13 knock-out C57Bl/6 mice. Inflammation and fibrosis were scored by macroscopic and histological examination and fibrosis was quantified by hydroxyproline. Numbers of Tregs and IFN-γ^+^, IL-13^+^ and IL-17A^+^ CD4^+^ T helper (Th) cells in mesenteric lymph nodes increased during chronic DSS administration and mRNA for IFN-γ and IL-17 in the inflamed colon tissue was upregulated. However, antibody-mediated neutralisation of IFN-γ or IL-17A/F in a therapeutic setting had no effect on chronic intestinal inflammation and fibrosis. Antibody-mediated depletion of Tregs did not enhance fibrosis, nor did IL-13 deficiency have an effect on the fibrotic disease. These data argue against an important contribution of Tregs and of the cytokines IFN-γ, IL-13, IL-17A, IL-17F in the induction and/or control of fibrosis in this Crohn’s disease like murine model.

## Introduction

Inflammatory bowel diseases (IBD) are the result of a complex interaction between genetic factors, the environment, the gut microbiota and the immune system, but a clear picture of the molecular pathways involved, is still missing^1^. After the identification of the pathogenic role of T helper 1 (Th1) and T helper 2 (Th2) cells, also T helper 17 (Th17) cells have been shown to play an important role. The Th1/Th2 paradigm in Crohn’s disease (CD) was subsequently revised and CD is currently seen as a Th1/Th17 driven disease^2^. Th17 cells have both pathogenic and protective functions during inflammation and infection^3^. To explain the increased recruitment and activation of different T cell subsets, a loss of regulatory control has also been implicated. CD4^+^ Foxp3^+^ T regulatory cells (Tregs) play a role in the maintenance of intestinal homeostasis, preventing pathological inflammatory responses to environmental factors and self-antigens. Treg transfer effectively suppresses experimental colitis and Tregs can suppress Th1- and Th2-mediated intestinal inflammation^4–9^. In 2012, Desreumaux *et al*. reported the first clinical trial using Tregs in the treatment of patients with CD. However, although promising, no more clinical studies were performed^10^.

Upon chronic inflammation, many CD patients develop fibrotic changes in the gut, leading to stricture formation and obstruction. The early stages of fibrosis are characterized by immunologic-inflammatory changes and an imbalance of anti- and pro-fibrotic cytokine profiles, leading to the generation of activated myofibroblasts, producing excessive extracellular matrix proteins. Cells and cytokines of the adaptive immune system have been suggested to play a prominent role in the initiation and progression of fibrosis. Th1 and Th2 cytokines play opposing roles in fibrosis: Th2 cytokines interleukin (IL-) 4 and IL-13 are implicated in fibrosis, whereas the Th1 cytokines interferon gamma (IFN-γ) and IL-12 suppress the development of tissue fibrosis^11^. In contrast, tumour necrosis factor (TNF) is a potent inducer of strictures in CD, although anti-TNF treatment has limited effects on remodelling in the gut^4,5,11^. The exact role of Th17 cells and IL-17 in the development of fibrosis needs further clarification^12^. Biancheri *et al*. reported that IL-17 is overexpressed in intestinal strictures in CD, while myofibroblasts express IL-17A receptors, produce more collagen and have reduced migratory ability in response to IL-17A^13^. As such, IL-17A might be important in promoting intestinal fibrosis. Moreover, recently, identification of innate lymphoid cells (ILCs) as important sources of Th1 and Th17 cytokines complicate the picture^6,14^. A better understanding of the roles of cytokines in fibrotic processes may reveal new pathways relevant for its pathogenesis and give more insights in the clinical observation that current anti-inflammatory strategies do not have effects on the prevention of fibrosis. Novel therapies, potentially able to induce a shift from a pro-fibrotic microenvironment to an anti-fibrotic microenvironment, may emanate from this research.

Previously, we have shown that repeated cycles of administration of DSS for one week followed by a recovery phase of two weeks in C57BL/6 mice, induce a relapsing and remitting disease course with transmural inflammation and typical connective tissue changes which resemble those of human CD^15^. This is therefore an easy-to-induce model of CD mimicking the human disease course. Melgar *et al*. demonstrated that a single exposure to DSS can progress to a chronic colitis in C57BL/6 mice with increased colonic expression of T cell derived cytokines^16^. Although multiple cycles of DSS induce chronic intestinal inflammation, little attention has been given to tissue remodeling in response to DSS^17–19^. Concerning the pathogenesis of DSS colitis, it is generally accepted that epithelial damage results in an influx of luminal bacterial products into the gut wall leading to acute colitis. However, the precise mechanisms underlying fibrogenesis in chronic murine (DSS) colitis are largely unknown.

The aim of this study was to investigate the involvement of Th1/Th2/Th17 type cytokines on intestinal fibrosis as well as the regulatory function of Tregs in chronic DSS colitis. Interventions were set up after initiation of disease, in order to mimic the clinical situation in CD patients, in whom anti-inflammatory therapy is also started after diagnosis in the chronic stage of the disease.

## Materials and Methods

### Mice and induction of colitis

**Figure 1:**
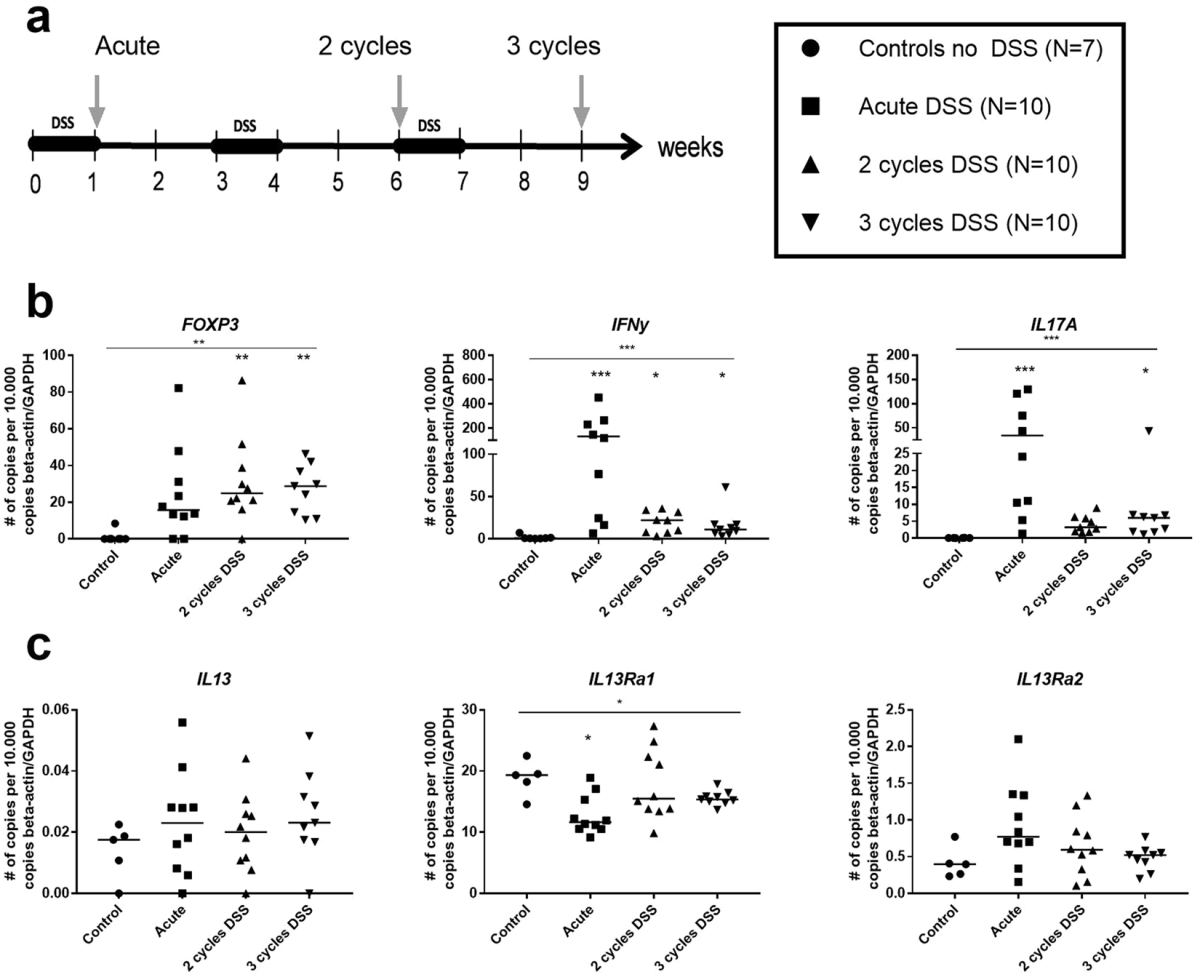
Colonic gene expression of FOXP3, IFN-y, IL17A/F, IL-13, IL-13Rα1 and IL-13Rα2. Mice were exposed to one (acute), two (2 cycles) or three (3 cycles) cycles of DSS and results were compared to unexposed controls. (**a**) DSS induction scheme. Arrows depict time of analysis. (**b**) *FOXP3*, *IFNy and IL17A* colonic mRNA level are measured by RT-PCR after 2 and 3 cycles of DSS. (**c**) *IL-13*, *IL-13Rα1* and *IL-13Rα2* mRNA levels in colon tissue measured by RT-PCR. Data are expressed as individual values and medians. Statistical analysis by Kruskal-Wallis testing for comparison of multiple groups. Multiple comparison with controls is shown by asterix above the group, multiple comparison between different groups are shown by horizontal lines above the groups. (*p < 0.05, **p ≤ 0.01, ***p ≤ 0.001).

To induce DSS colitis, female 6-weeks old C57BL/6OlaH mice were obtained from Envigo (Horst, The Netherlands). IL-13 knock-out (KO) mice were originally generated in the laboratory of GJ McKenzie and backcrossed on the C57B/6 background by Prof Hoshino T^20,21^. During the experiments, all animals were maintained in the Animal Care Facility of the Faculty of Medicine, University of Leuven (Belgium). Colitis was induced as previously described^15^. Briefly, 1.5–2.0% DSS (35–50 kDa; MP Biomedicals, Illkirch, France) was added to the drinking water to induce colitis. Acute colitis mice received DSS for 7 days. For the study of chronic colitis, mice were exposed to repeated “cycles” of DSS exposure. One cycle was defined as exposure to DSS for 7 days followed by a recovery period of two weeks with normal drinking water (Fig. 1a). Mice were exposed to one, two or three cycles in total. Control mice received normal drinking water. As all experiments were performed in the same timeframe, only one control group was added. All mice were age-matched at the time of sacrifice.

### Evaluation of inflammation and fibrosis

Animals were euthanized with sodium pentobarbital (Nembutal, Ovation Pharmaceuticals Inc. Deerfield, US) and the inflammation in distal colon was evaluated as previously described^18^. The Disease Activity Index (DAI) was determined based on body weight loss, stool consistency and presence of gross blood in the stools. A macroscopic damage score was calculated on the base of the presence of adhesions, hyperemia and extent of colonic inflammation. Histology was performed on paraffin embedded, 5 µm-thick longitudinal and transverse sections stained with hematoxylin and eosin. The microscopic score of inflammation was calculated as previously described^18^. Briefly, this score comprised the sum of changes in mucosal architecture, neutrophil infiltration, mononuclear cell infiltration, goblet cell loss and epithelial defects. The histological active disease score comprised the sum of neutrophil infiltration and epithelial defects, reflecting the activity of disease and the potential for acute tissue damage during the last 24 hours prior to tissue sampling. Slides were scored by an experienced pathologist (KG, GdH) blinded to the experimental condition. Fibrosis was evaluated by hydroxyproline quantification and Martius Scarlett Blue stainings as previously described^15^. In short, areas stained for collagen in the mucosa and submucosa were measured per cross-section using ImageJ^20^. The thickness of the mucosa and muscularis propria was calculated as mean value of two different measurements per mouse on uniform horizontal cross sections of colon crypts using ImageJ^22^. A hydroxyproline assay was performed as previously described^23^.

### Anti-cytokine antibody treatments

Antibodies or corresponding isotype controls, diluted in sterile PBS, were administered intraperitoneally (ip) once weekly (0.5 mg/mouse/week) during the second and the third cycle of DSS administration (from day 21 until day 63). The PC 61 cell clone (Rat anti-mouse CD25 IgG_1_, #TIB-222) was obtained from ATCC (Manassas, VA)^24^. Rat IgG_1_ HRPN (cat #BE0088), anti-mIFN-γ (XMG1.2, cat #BE0055) and mouse IgG_1_ (MOPC-21, cat #BE0083) were obtained from BioXCell (West Lebanon, NH)^25–27^. Mouse anti-mIL-17A (MM17F3) and mouse anti-mIL-17F (MM17F-8FS) were generated and kindly provided by Jacques Van Snick and Catherine Uyttenhove (Ludwig Institute for Cancer Research, Brussels, Belgium)^28,29^. In control mice, not exposed to DSS, the corresponding volume of sterile PBS was administered.

### Flow cytometry

One hundred µl of whole blood in EDTA, was stained with anti-CD4-PerCP-Cy5.5 (eBioscience, San Diego, CA, cat #45-0042) and anti-CD25 PE (eBioscience, cat #12-0251). After incubation for 30 minutes, red blood cells (RBC) were lysed using freshly prepared RBC lysis buffer (0.1 mM EDTA, 155 mM NH4Cl, 10 mM KHCO3 in sterile water). For intracellular detection of Foxp3 (anti-Foxp3 AF 488, eBioscience, clone FJK-16s), the protocol guidelines of the Foxp3 staining kit (eBioscience, cat #00-5523-0) were followed. After washing, cells were fixed in 1% paraformaldehyde. Mesenteric lymph nodes (MLNs) lymphocytes were further stained with anti-Foxp3, anti-CD45 brilliant violet (BV) 510 (Biolegend, clone 30-F11), anti-CD3-PE-Cy7 (eBioscience, clone 7D6) and anti-CD4-PerCP-Cy5.5 (eBioscience, clone RM4-5) as previously described. For intracellular cytokine detection MLNs lymphocytes were stimulated with phorbol myristate acetate 100 ng/ml, ionomycin 1 µg/ml (Sigma) and monensin 0.7 µg/ml (Sigma) followed by anti-CD4-PerCP-Cy5.5 staining. Cells were fixated and washed with permeabilisation buffer (0.5% saponin, 0.5% BSA in PBS) before staining with anti-IFN-γ PE (eBioscience, cat #12-7311-82) or anti-IFN-γ FITC (BD, Erembodegem, Belgium, clone XMG1.2), anti-IL-17A PE (BD, clone TC11-18H10) or anti-IL-17A AF488 (BD, clone TC11-18H10) or anti-IL-13 PE (eBioscience, clone eBio13A) if applicable. Fluorescence minus one (FMO) controls were included in all experiments. Analysis was performed using the BD Cellquest software on a FACSort flow cytometer (BD).

### Quantitative RT-PCR analysis

Total RNA was extracted from snap frozen colon (Qiagen RNeasy Mini Kit cat #74106). A fixed amount of target RNA (1 µg) was reverse transcribed using 500U MultiScribe® Reverse Transcriptase (ThermoFisher) at 37 °C for 120 min. Real-time RT-PCR was performed for murine *ACTA-2*, *COL1A1*, *IL-13*, *IL13Ra1*, *IL13Ra2*, *IL-17*, *IFN-y* and *FoxP3* mRNA in a CFX Connect™ Real-Time PCR Detection System (Bio-Rad, Nazareth, Belgium). Murine *β-actin* and *GAPDH* were used as housekeeping genes. PCR amplification for each sample was performed in duplicate wells.

### Statistical analysis

Statistical analysis and calculations were performed using GraphPad Prism 7 (GraphPad, La Jolla, CA, USA) and R (version 2.7.2, http://www.r-project.org). Data are represented as medians and the individual p-values for two groups comparison were obtained using Mann-Whitney U testing. P-values for comparison between multiple groups were obtained using Kruskal-Wallis testing with Dunn’s multiple comparison. Differences were considered statistically significant at p < 0.05. Significance levels (in comparison to control mice without administration of DSS) are shown above each group if applicable (*p < 0.05, **p ≤ 0.01, ***p ≤ 0.001).

### Ethical considerations

All studies were approved by and performed according to the local ethics committee for animal experimentation of the University of Leuven (P024-2010 and P134-2010).

## Results

### Effects of repeated cycles of DSS on colonic cytokine mRNA levels

We previously reported that repeated cycles of DSS administration for one week to C57Bl/6 mice followed by a recovery phase of two weeks (see Fig. 1a), induced a relapsing and remitting disease course with transmural inflammation and typical connective tissue fibrotic changes^15^. To study the mechanism behind the induction of fibrosis, we first performed additional analysis of tissue mRNA on samples from these previously published experiments^15^. Microarray analysis of colonic tissue after induction of acute DSS colitis and after two or three cycles of DSS exposure showed upregulation of mRNA of Th1 and Th17 cytokines including *IFN-y* and *IL-17* after each cycle as compared to controls (data no shown). Increased levels of *IL-17A*/*F* (p < 0.001), *IFN-y* (p = 0.001) and *FoxP3* (p < 0.001) mRNA were confirmed by RT-PCR on colonic tissue after 3 cycles of DSS exposure as compared to control mice (Fig. 1b). No significant differences in expression level of *IL-13* mRNA between the different experimental groups were observed. In acute DSS colitis and after 1 cycle of administration of DSS, *IL-13RA1* mRNA expression was lower than in the controls, but normalized after further DSS cycles. No difference in the colonic expression of *IL-13RA2* mRNA in comparison to control colon was seen (Fig. 1c).

### Effects of repeated cycles of DSS on T helper subsets and T regulatory cells in blood and MLN

**Figure 2:**
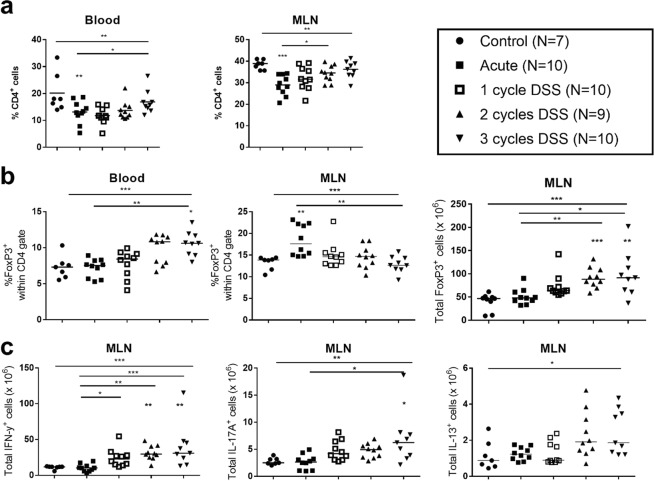
Analysis of CD4^+^ and Foxp3^+^CD4^+^ lymphocytes in blood and mesenteric lymph nodes. Flow cytometric analysis of blood and MLN cells was performed in mice exposed to DSS in the acute model and after two or three cycles of DSS exposure. (**a**) Relative proportions of CD4^+^ T cells within the lymphocyte gate in whole blood and in mesenteric lymph nodes (MLN). (**b**) Proportion of Foxp3^+^CD4^+^ Tregs within the CD4^+^ gate in whole blood and MLN. (**c**) Total number of IFN-γ, IL-17A and IL-13 expressing CD4^+^ T cells in MLN (after *ex vivo* PMA and ionomycin stimulation). Control mice N = 7, acute colitis N = 10, 1 cycle colitis N = 10, 2 cycle colitis. N = 10, 3 cycle colitis N = 9. Data are expressed as medians and individual values. Kruskal-Wallis testing is shown over all groups. Multiple comparison with controls is shown above the group, multiple comparison between different groups are shown by horizontal lines above the groups. (*p < 0.05, **p ≤ 0.01, ***p ≤ 0.001).

To explore whether repeated administration of DSS also has systemic effects, we examined Th1, Th2, Th17 and CD4^+^Foxp3^+^ Tregs in blood and MLNs in animals with acute or chronic DSS colitis. Both in blood and MLNs, a significantly lower percentage of CD4^+^ cells within the lymphocyte gate was observed in mice with acute DSS colitis (p = 0.033 and p < 0.001 respectively compared to control mice). However, during repeated cycles of DSS, the proportion of CD4^+^ T cells increased progressively and returned to normal percentages after the third cycle of DSS (Fig. 2a). In blood, a significant increase was seen in the percentage of CD4^+^Foxp3^+^ Tregs within the lymphocyte gate after 2 and 3 cycles of DSS administration (p = 0.01, p = 0.001 respectively as compared to controls) (Fig. 2b). In MLNs, a significant higher percentage of CD4^+^Foxp3^+^ Tregs was observed within the lymphocyte gate already in the acute colitis phase (p < 0.001). The total number of CD4^+^Foxp3^+^ cells in MLNs further increased significantly with more cycles of DSS administration (p < 0.001) (Fig. 2b). Upon intracellular cytokine staining (Suppl. Fig. S1) we observed a significant increase in total numbers of IFN-γ^+^, IL-13^+^ and IL-17A^+^ CD4^+^ T cells in the MLN with increasing cycles of DSS (p < 0.001, p = 0.015, p = 0.001 respectively) (Fig. 2c). Altogether, these results illustrate the induction of a systemic Th1, Th2, Th17 and Treg response in chronic DSS colitis.

### Inhibition of T regulatory cells enhances systemic and colonic inflammation

**Figure 3:**
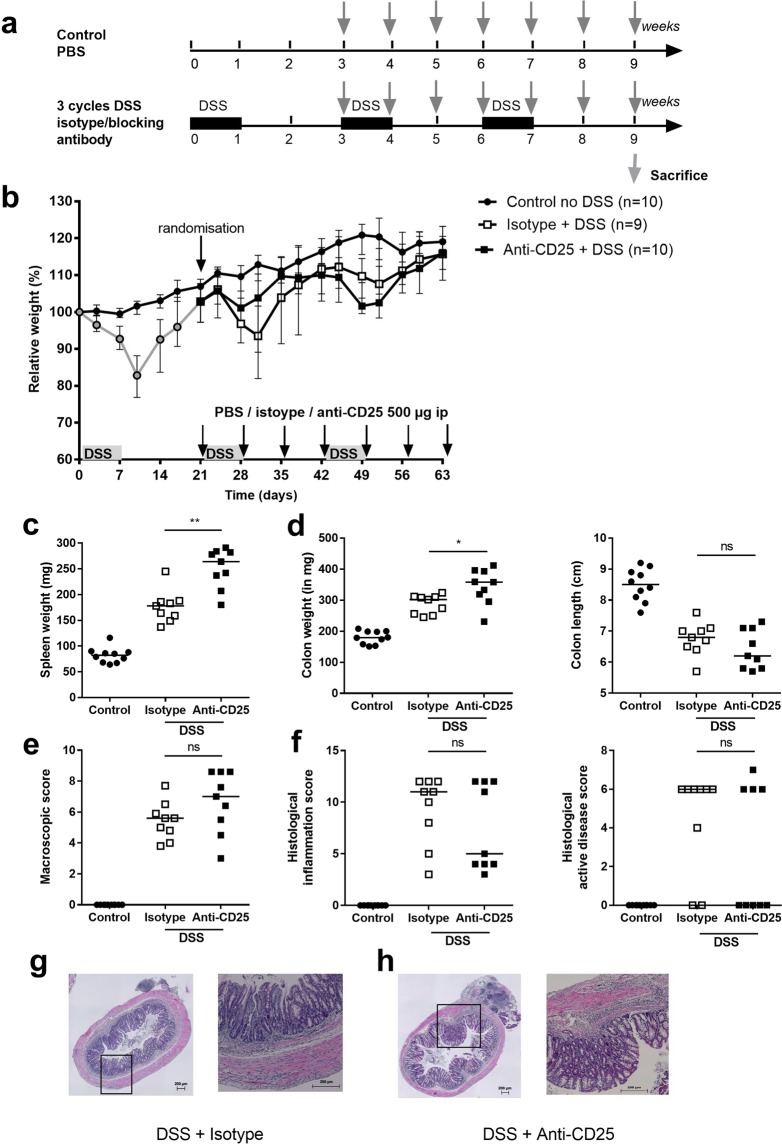
Effects of anti-CD25 administration in chronic DSS colitis. (**a**) Experimental design of the experiments with the administration of inhibitory antibodies. Three cycles of DSS administration were performed to induce colitis, and effects were compared to non-DSS exposed control WT mice. Anti-CD25 administration started after the first cycle of DSS. Grey arrows depict anti-CD25 administration. (**b**) Relative weight curve (rat IgG1 anti-CD25 group: N = 10, isotype IgG1 group: N = 9, control mice without DSS administration: N = 10). (**c**) Spleen weight. (**d**) Colon weight and colon length. (**e**) Macroscopic damage score. (**f**) Histological inflammation score and histological active disease score. Per mouse, the mean score of two cross-sections and one longitudinal section of each of the five parameters was calculated. Representative HE staining from isotype (**g**) and anti-CD25 administration (**h**) left 10×, right 40× magnification. Data are expressed as medians with individual values. Mann-Whitney U testing (*p < 0.05, **p ≤ 0.01, ***p ≤ 0.001).

Next, we studied the effect of blocking Tregs by anti-CD25 administration during the second and third cycle of administration of DSS (Fig. 3a)^24^. Efficient reduction of Foxp3^+^ cells after anti-CD25 treatment as compared to controls (2.67 vs 7.37% of CD4^+^ T cells, p < 0.01) was achieved (Suppl. Fig. S2). Although no significant differences in relative body weight (Fig. 3b) were observed, spleen weight (p = 0.002), colon weight (p = 0.024) and colon weight/length ratio (p = 0.030) were significantly higher in the anti-CD25 group compared to isotype treated mice (Fig. 3c,d). Colon length, macroscopic damage scores and histological inflammation scores were not significantly different in both groups (Fig. 3d–h).

### Inhibition of T helper cell associated cytokines does not alter systemic and colonic inflammation

**Figure 4:**
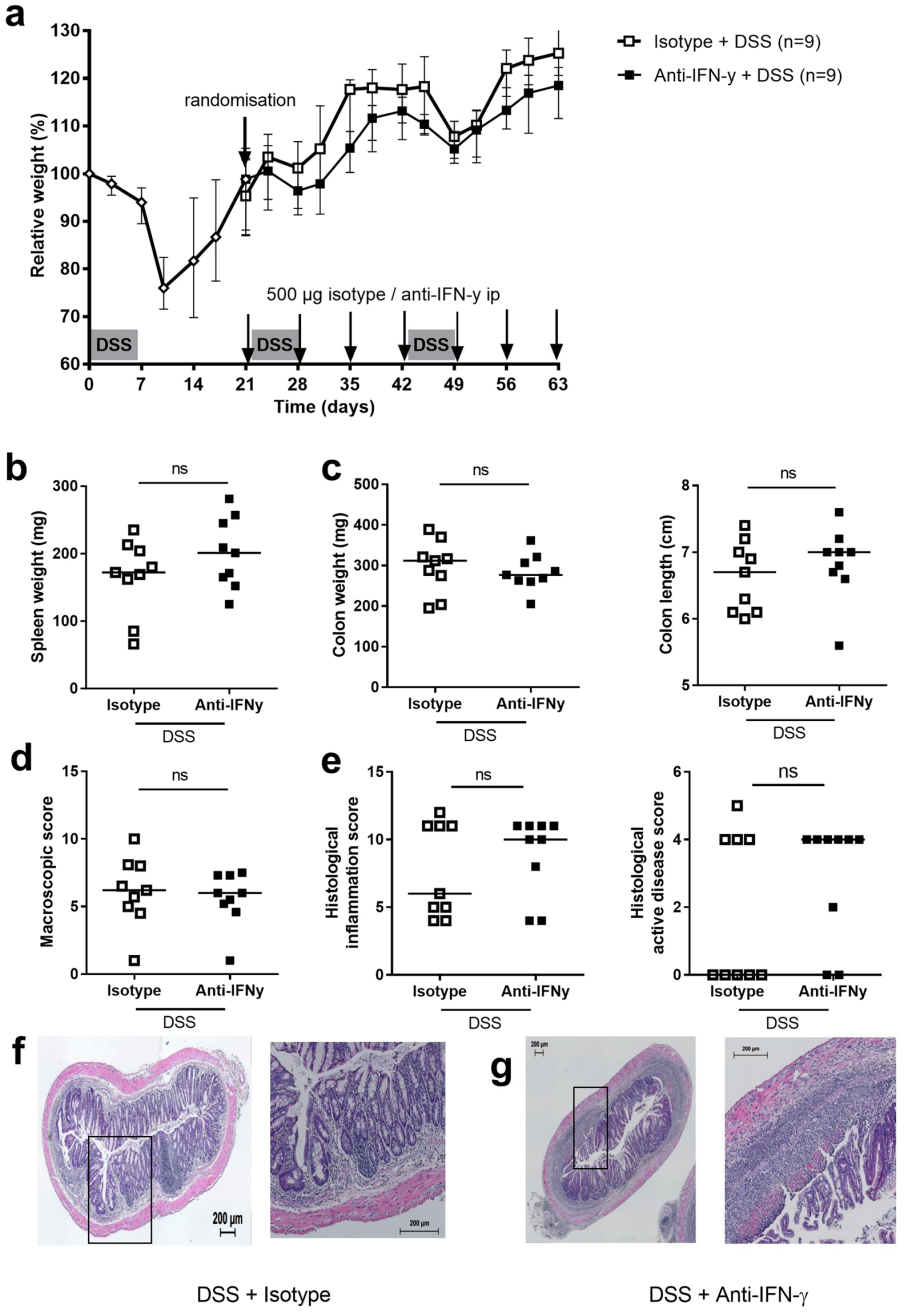
Effect of anti-IFN-γ administration in chronic DSS colitis. At day 21, after cycle 1, all DSS exposed mice were randomized to equal relative weight in both groups. Anti-IFN- γ and isotype administration started after the first cycle of DSS. During the second cycle of administration of DSS, one mouse died in the anti-IFN-γ group. The control group (not exposed to DSS) is not shown but us the same as in Fig. 3. (**a**) Relative weight curve (mouse IgG_1_ anti-IFN-ɣ group: N = 9, isotype IgG_1_ group: N = 9). (**b**) Systemic inflammation: spleen weight. (**c**) Colon read-outs: colon weight and colon length. (**d**) Macroscopic damage score. (**e**) Histological inflammation score and histological active disease score. Representative HE staining for isotype (**f**) and anti-IFN-γ administration (**h**) left 10×, right 40× magnification. Data are expressed as medians with individual values. Mann-Whitney U testing (*p < 0.05, **p ≤ 0.01, ***p ≤ 0.001).

**Figure 5:**
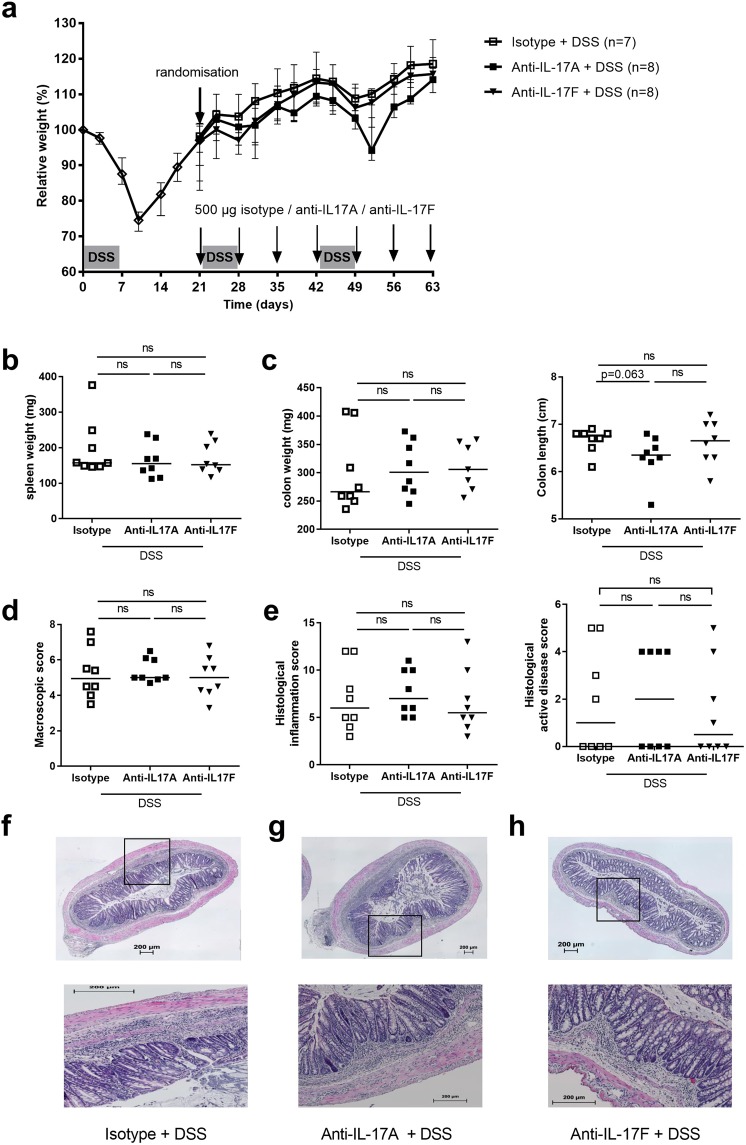
Effects of anti-IL-17A and anti-IL-17F administration in chronic DSS colitis. At day 21, after one cycle of DSS, all DSS exposed mice were randomized. (**a**) Relative weight curve (mouse IgG1 anti-IL-17A group: N = 8, mouse IgG_1_ anti-IL-17F group: N = 8, isotype IgG_1_ group: N = 7). The control group (not exposed to DSS) is not shown but is the same as in Fig. 3. (**b**) Spleen weight. (**c**) Colon read-outs: colon weight and colon length. (**d**) Macroscopic damage score. (**e**) Representative HE staining for isotype treated (**f**), anti-IL-17A (**g**) or anti-IL-17F (**h**) treated groups; top 10×, bottom 40× magnification. Histological inflammation score and histological active disease score. Data are expressed as medians with individual values. Mann-Whitney U testing (*p < 0.05, **p ≤ 0.01, ***p ≤ 0.001). Uncorrected p-values are shown.

We then investigated the effect of inhibition of IFN-γ on chronic inflammation by administration of anti-IFN-γ antibodies in a therapeutic setting (i.e. administration during the second and third cycle) (Fig. 4)^25–27^. Mice receiving anti-IFN-γ during the second and third cycle of DSS had a lower relative body weight compared to isotype-treated mice (Fig. 4a). However, no differences were observed in spleen weight, DAI, colon weight, colon length and macroscopic damage score after administration of anti-IFN-γ *vs* isotype (Fig. 4b–d) nor histological inflammation score and histological active disease (Fig. 4e–g). Next, we studied the effect of inhibition of IL-17A or IL-17F with mouse anti-mouse antibodies in a similar setting (Fig. 5)^29–32^. Although mice receiving anti-IL-17A tended to lose more weight compared to isotype control mice and to mice receiving anti-IL-17F (Fig. 5a), no differences were observed on the level of spleen weight or colon read-outs nor on microscopic inflammation (Fig. 5b–g).

### Administration of anti-IL17A induces a slight increase in collagen deposition in the colon

**Figure 6:**
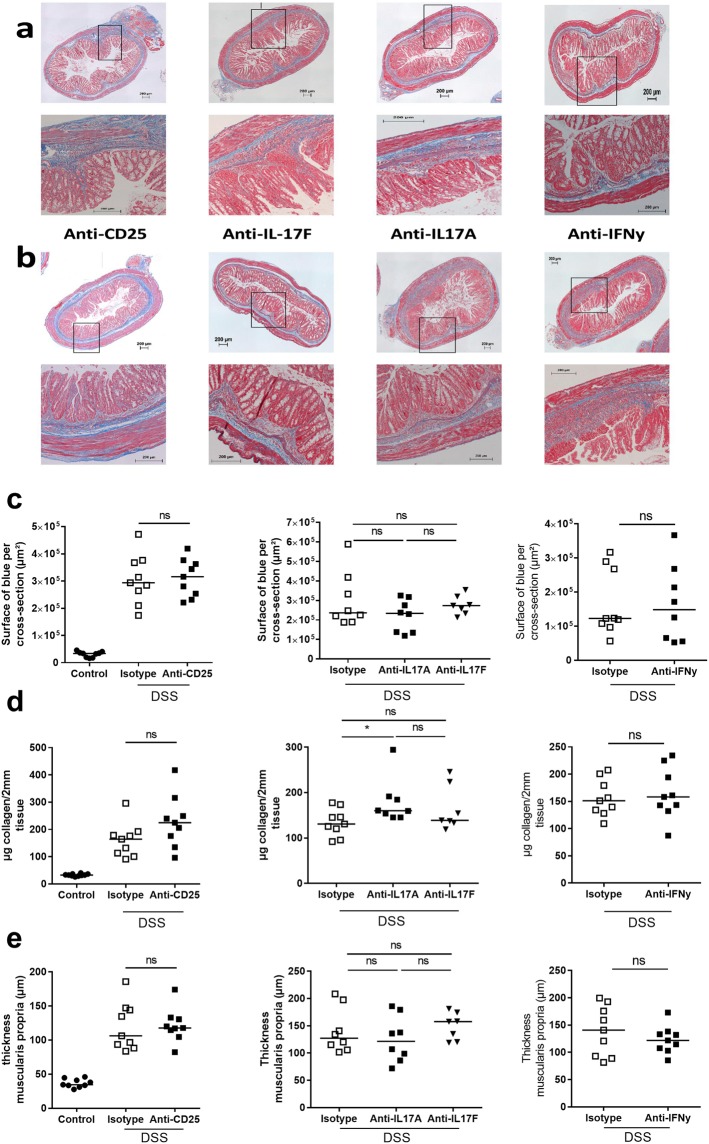
Connective tissue changes after administration of cytokine inhibitory antibodies or of anti-CD25. Experimental set-up as explained in Fig. 4. Analysis was performed after 3 cycles of DSS. (**a**,**b**) Representative pictures of a MSB trichrome staining after administration of (**a**) isotype IgG and (**b**) anti-CD25 (PC61), anti-IL17A (MM17F3), anti-IL17F (MM17F-8FS) and anti-IFN-ɣ (XMG1.2) at the end of 3-cycles DSS colitis exposure with recovery phase. Scale bar 200 µm. (**c**) Quantification of the area of fibrosis in MSB trichrome staining in healthy controls or after anti-CD25, anti-IFNy, anti-IL17A or anti-IL17F treatment, (**d**) hydroxyproline quantification, (**e**) thickness of the muscularis propria. Per animal, two sections were evaluated. Data are expressed as medians with individual values. Mann-Whitney U testing (*p < 0.05, **p ≤ 0.01, ***p ≤ 0.001). Uncorrected p-values are shown.

Effects of depletion of Tregs by anti-CD25 administration, and of administration of anti-IFN-γ, anti-IL-17A or anti-IL-17F on fibrosis after three cycles of DSS, were evaluated first by quantification of collagen deposition in mucosa and submucosa by MSB trichrome staining (Fig. 6a–c). No differences in MSB staining or in the level of collagen in the colonic wall (hydroxyproline assay) were observed after neutralization of IFN-ɣ or IL-17F or elimination of Tregs (Fig. 6a–d) nor in the thickness of the muscularis propria as compared to isotype treated mice (Fig. 6e). DSS colitis resulted in a significant increase of *Col1A1* gene expression at the mRNA level, but this was also not affected by treatment (Suppl. Fig. S3) Surprisingly, inhibition of IL-17A was associated with an increase in collagen concentration as compared to isotype (p = 0.027), with also a tendency to an increase on the mRNA level, but no effect was seen in MSB staining.

### No attenuation of inflammation and fibrosis in IL-13 deficient mice

**Figure 7:**
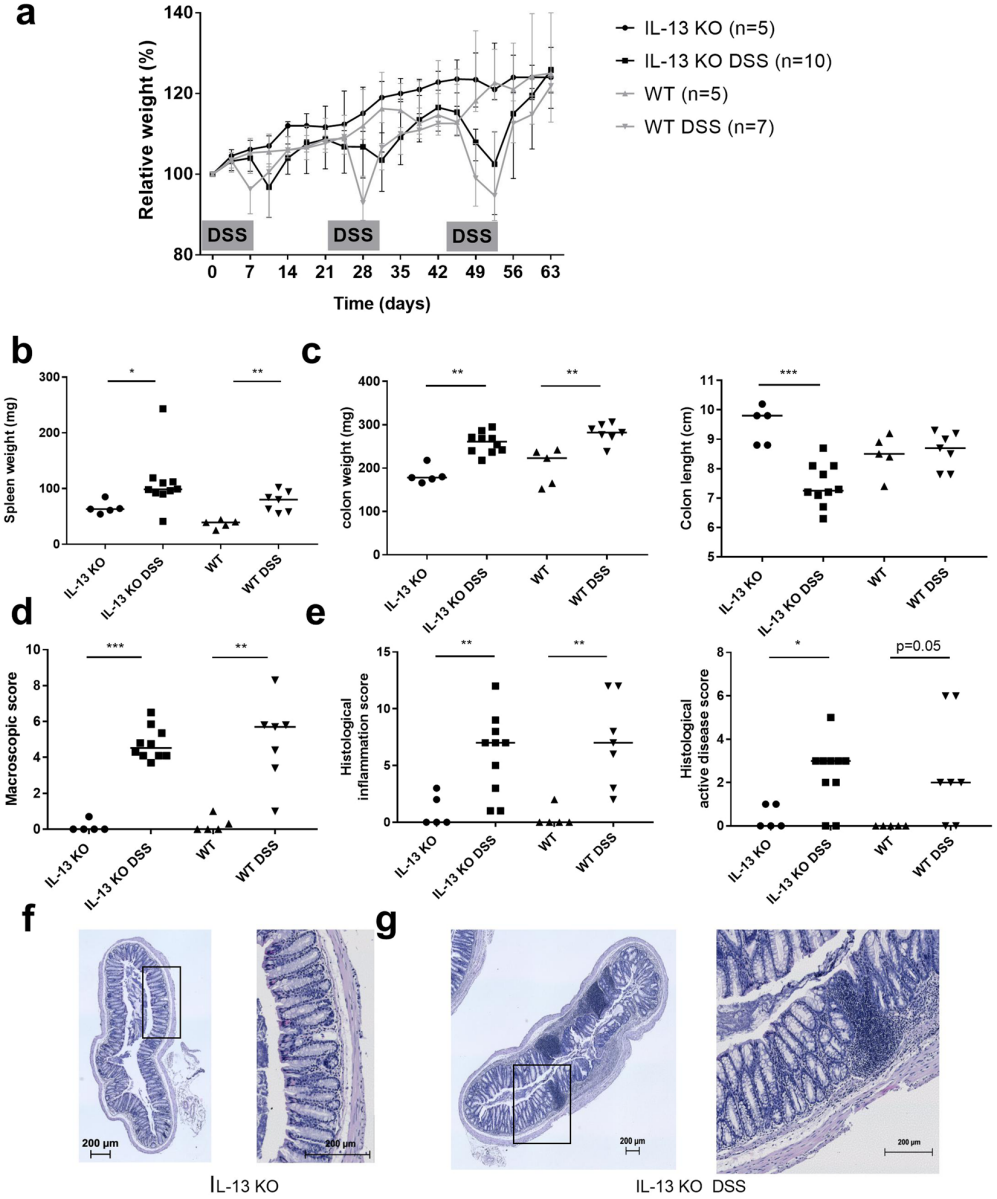
Induction of chronic DSS colitis in IL-13 KO mice. (**a**) Relative weight curve of IL-13 KO controls and IL-13 KO after induction of chronic DSS colitis as compared to WT mice with or without DSS. (**b**) Spleen weight. (**c**) Colon weight and colon length. (**d**) Macroscopic damage score. (**e**) Histological inflammation score and histological active disease score. Representative HE staining from control IL-13 KO (**f**) and chronic DSS exposed IL-13 KO mice (**g**) with clear granuloma formations; left 10×, right 40× magnification. Data are expressed as medians with IQR. Mann-Whitney U testing (*p < 0.05, **p ≤ 0.01, ***p ≤ 0.001).

**Figure 8:**
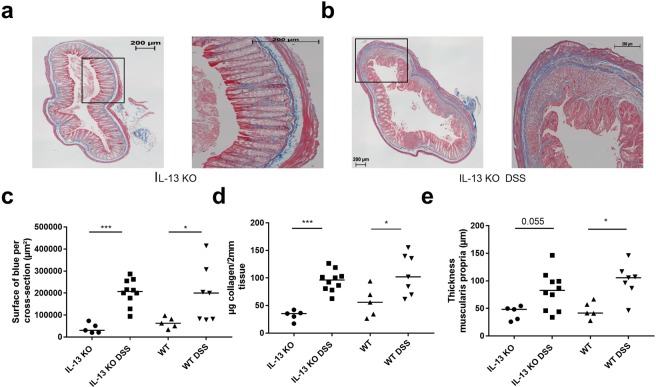
Connective tissue changes after chronic DSS colitis induction in IL-13 deficient mice. (**a**,**b**) Representative pictures of a MSB trichrome staining in age-matched IL-13 KO control (**a**) and IL-13 KO chronic colitis mice (**b**) at the end of 3-cycles DSS colitis. Scale bar 200 µm. (**c**) Quantification of the area of fibrosis in MSB trichrome staining, (**d**) hydroxyproline quantification, (**e**) thickness of the muscularis propria of IL-13 KO mice as compared to WT mice. Per animal, two sections were evaluated. Data are expressed as medians and individual values. Mann-Whitney U testing (*p < 0.05, **p ≤ 0.01, ***p ≤ 0.001). Uncorrected p-values are shown.

Next, we studied the role of IL-13 in a chronic DSS colitis in IL-13 KO mice (Fig. 7a)^20,21^. Similar wasting disease was induced in IL-13 KO mice by repeated DSS without attenuation of inflammation based on DAI, macroscopic and microscopic score (Fig. 7a–g). Quantification of collagen deposition in mucosa and submucosa by MSB staining revealed increased collagen deposition (Fig. 8a–d) and an increased thickness of the muscularis propria in DSS exposed IL-13 KO mice as compared to IL-13 KO controls (Fig. 8e). Furthermore, collagen concentrations were elevated on both protein (Fig. 8d) and mRNA level (Suppl. Fig. S4) in chronic DSS exposed IL-13 KO mice. These effects of DSS exposure were similar to the ones observed in WT mice.

## Discussion

The aim of this study was to evaluate the role of the major Th1, Th2 and Th17 cytokines in the chronicity of inflammation and fibrosis in chronic DSS colitis, and whether Tregs have a modulatory effect in this model. We showed the progressive induction of systemic Th1, Th12, Th17 cells and Tregs in blood and MLNs with increasing number of cycles of DSS. Next, we showed that reduction of Tregs in a therapeutic setting enhanced systemic inflammation (spleen weight) but had only minimal effects on colonic inflammation and no effect on fibrosis. While inhibition of IFN-γ or IL-17F activity had no effect neither on histological colonic inflammation nor on the deposition of collagen in the colon, inhibition of IL-17A was associated with a slight but significant increase in collagen deposition, however without affecting colonic or systemic inflammation. Last, we could show that in absence of IL-13, DSS colitis with intestinal remodeling and fibrosis could still be induced in a similar manner as in WT animals.

Previously, this chronic DSS model with relapse and remission was developed in order to mimic the disease course of human CD^15,33^. Induction of fibrosis in chronic DSS colitis was only observed after at least 2 cycles of DSS administration during one week, each followed by a recovery period of 2 weeks^15^. In the present experimental set-up we started treatment with inhibitory antibodies only from the second cycle of DSS administration onwards in a pre-clinical approach. We indeed intended to avoid interfering with the acute inflammatory phase, and to specifically influence the induction of connective tissue changes during the chronic phase (reflecting clinical reality). In addition, previous studies have already addressed the effects of neutralising antibody treatments in the early phase of DSS induced colitis^34–37^.

The identification of a decrease in Foxp3^+^ expressing Tregs in peripheral blood and MLN in acute colitis followed by a recovery phase and increase above normal levels in chronic DSS colitis are in line with the findings in human IBD^38^. Treg numbers are increased in the mucosa of patients with IBD as a consequence of ongoing inflammation^39,40^. Probably, this results from recruitment to or generation of Foxp3^+^ Tregs in the intestinal mucosa as a result of ongoing inflammation and cytokine production. A relative functional deficiency of these cells is supposed to result in incomplete control of inflammation^40–43^. Previous reports showed that intermittent depletion of Foxp3^+^ Tregs aggravates intestinal inflammatory responses in acute DSS colitis and in the transfer model^25^. Despite more severe systemic inflammation, due to downregulation of Tregs, this does not seem to alter intestinal fibrosis, demonstrating the complex interplay between Foxp3^+^ Tregs and mucosal immunity in the intestine.

The role of IL-13 in IBD is debated. IL-13 has been implicated in UC and in other fibrotic diseases including idiopathic pulmonary fibrosis and liver fibrosis but the knowledge of the role of IL-13 in fibrosis in CD is limited^44–46^. Biancheri *et al*. could not show different levels of IL-13 in strictured and non-strictured UC and CD explants as compared to controls^44^. These data are in line with our findings that fibrosis can still be induced in IL-13 deficient mice indicating a limited role of IL-13 in intestinal fibrosis in IBD. On the other hand, elevated levels of *IL-13* mRNA could be detected in the colonic muscle wall from fibrotic CD patients^47^. Biologicals targeting IL-13 in UC showed different success rates; anrukinzumab had no therapeutic effect in UC, while tralokinumab was beneficial for only some patients with UC^44,46^. In these studies the effect on long-term fibrosis was not studied.

Inhibition of IFN-γ, IL-17A or IL-17F during the chronic phase of colitis had no clear effect on chronic colonic or systemic inflammation^35–37^. Previous data showed that IFN-γ^−/−^ mice did not develop colitis after administration of DSS^26^. mRNA colonic expression levels of *IFN-γ* indicate an important production in early, acute inflammation while production is decreased in the chronic phase of inflammation^35^. These results are not necessarily contradictory to ours, because we started anti-IFN-γ treatment after the onset of inflammation. An anti-fibrotic role has been suggested for IFN-γ, but we were not able to show an effect of IFN-γ neutralization on collagen deposition in this experimental set-up. Clinical studies investigating the therapeutic potential of IFN-γ have also been disappointing^48^.

IL-17A has been implicated in pulmonary, myocardial and hepatic fibrosis^49–51^. IL-17A has been shown to directly induce expression of MMP-1 in human cardiac fibroblasts, suggesting that IL-17A promotes fibrosis by both exacerbating the upstream inflammatory response and regulating the downstream activation of fibroblasts^52^. Moreover, recent data suggested a role of IL-17A in fibrogenesis in CD^13,53^. *In vitro*, IL-17A could induce epithelial-to-mesenchymal transition (EMT) in intestinal epithelial cells, while *in vivo* IL-17A neutralization in chronic TNBS could attenuate intestinal fibrosis through inhibition of EMT^53^. Lo BC *et al*. showed innate IL-17A mediated gut fibrosis in a chronic Salmonella model^54^. However, IL-17 has both pathogenic and protective functions during inflammation and the timing of neutralisation might therefore be critical^55,56^. Our finding on increased deposition of extracellular matrix after administration of inhibitory antibodies to IL-17A is an interesting finding however only demonstrated with hydroxyproline assay and not confirmed with MSB staining. Validation and confirmation of these results is required. However these data contradict with findings in lung and hepatic fibrosis where IL-17A neutralization resulted in an amelioration of fibrosis^57,58^. Moreover, fontolizumab, an anti-IFN-γ antibody, and both secukinumab and brodalumab, targeting IL-17A and IL-17R respectively, failed to show clinical benefit in the treatment of CD^59–62^.

One limitation of this study is that the effect of neutralization of TNF was not tested. Studies in rats could show attenuation of fibrosis after vehicle-mediated TNF neutralization in peptidoglycan-polysaccharide colitis^63^. Although therapeutic TLA1 neutralization ameliorated fibrosis in a transfer model of colitis, it was not as effective in a setting of preventive neutralization^64^. Furthermore, poor outcome efficacy was seen with infliximab on intestinal fibrosis in patients with IBD, and therapy failure was shown to be associated with subclinical fibrosis in Crohn’s disease^65^. Another limitation is that neutralisation with mAbs does not necessarily eliminate all cytokine activity. However, we used antibodies that were previously shown to be effective for cytokine neutralisation in other disease models. The anti-IFN-ɣ antibody XMG1.2 has been shown to have a clear effect of neutralization in literature. In a study by Mohr *et al*., administration of 1 mg of the XMG1.2 antibody twice with a three day interval blocked IgG isotype switch induced by Th1 cells^25^. In a study by Quezada *et al*. this antibody blocked the anti-tumor effect of CD4 cells when given at a dose of 200 µg per mouse 6 times over a 12 day period^26^. In a study by Prencipe *et al*., the antibody was given in a model of macrophage activation syndrome at a dose of 100 µg/g body weight, and was shown to block mortality^27^. The anti-IL17A and IL-17F mAbs are particular in the sense that they are mouse anti-mouse antibodies. We have previously shown that the anti-IL-17A mAb given at a dose of 200 µg/mouse twice weekly completely blocked EAE induction, and that the antibody was also effective in ICOS KO mice which have an exacerbated form of EAE due to higher IL-17 production^66^. Several other publications could show inhibitory effects of these same antibodies in a similar injection scheme. A convincing example is the publication by Chenuet, P. *et al*. showing that neutralization of either IL-17A or IL-17F is sufficient to inhibit house dust mite induced allergic asthma in mice^32^.

In summary, these results suggest that neither the main Th1, Th2 and Th17 cytokines nor Tregs, although all increased in chronic DSS colitis, are not crucial in the chronicity of inflammation and fibrosis. Our data do not exclude a major role of other Th cytokines and/or innate immune cells as main actors. To further unravel fibrogenesis in IBD, induction of chronic colitis should be studied in innate models of intestinal remodeling in the absence of adaptive immunity, with a focus on the role of macrophages and ILCs as potential sources of pro-fibrotic factors. More studies in this easy-to-induce model that mimics human CD can lead to the identification of new targets for intervention to meet the current therapeutic needs.

## Supplementary information

SUPPLEMENTARY FIGURES - FIBROGENESIS IN CHRONIC DSS COLITIS IS NOT INFLUENCED BY NEUTRALISATION OF REGULATORY T CELLS, OF MAJOR T HELPER CYTOKINES OR ABSENCE OF IL-13

## Data Availability

The datasets generated during and/or analysed during the current study are available in the Gene Expression Omnibus under the series Accession Number GSE42768, http://www.ncbi.nlm.nih.gov/geo/query/acc.cgi?token=ztwzpaseamwoora&acc=GSE42768.
