# Supplementary material for: Fibrogenesis in Chronic DSS Colitis is Not Influenced by Neutralisation of Regulatory T Cells, of Major T Helper Cytokines or Absence of IL-13
Source: Sci Rep. 2019 Jul 11;9:10064. doi: 10.1038/s41598-019-46472-6 (PMC6624199; doi:10.1038/s41598-019-46472-6)
Supplement: Supplementary file 1 — SUPPLEMENTARY FIGURES - FIBROGENESIS IN CHRONIC DSS COLITIS IS NOT INFLUENCED BY NEUTRALISATION OF REGULATORY T CELLS, OF MAJOR T HELPER CYTOKINES OR ABSENCE OF IL-13 [file 41598_2019_46472_MOESM1_ESM.pdf]

FIBROGENESIS IN CHRONIC DSS COLITIS IS NOT INFLUENCED BY NEUTRALISATION OF REGULATORY T CELLS, OF MAJOR T HELPER CYTOKINES OR ABSENCE OF IL-13

Brecht Creyns[1,2], Jonathan Cremer[1,2], Tomoaki Hoshino[3] , Karel Geboes[4], Gert de Hertogh[4], Marc Ferrante[2,5], Séverine Vermeire[2,5], Jan L Ceuppens [1], Gert Van Assche[2,5], *Christine Breynaert[1,6]

[1] KU Leuven, Department of Microbiology, Immunology and Transplantation, Allergy and Clinical Immunology Research Group, Leuven, Belgium.
[2] KU Leuven, Department of Chronic Diseases, Metabolism and Ageing, Translational Research Center for Gastrointestinal Disorders (TARGID), Leuven, Belgium.
[3] Division of Respirology, Neurology and Rheumatology, Department of Internal Medicine, Kurume University School of Medicine, Japan.
[4] KU Leuven, Department of Imaging and Pathology, Translational Cell & Tissue Research, Leuven, Belgium.
[5] University Hospitals Leuven, Department of Gastroenterology and Hepatology, Leuven, Belgium.
[6] University Hospitals Leuven, Department of General Internal Medicine, Leuven, Belgium.

SUPPLEMENTARY FIGURES

Supplementary figures

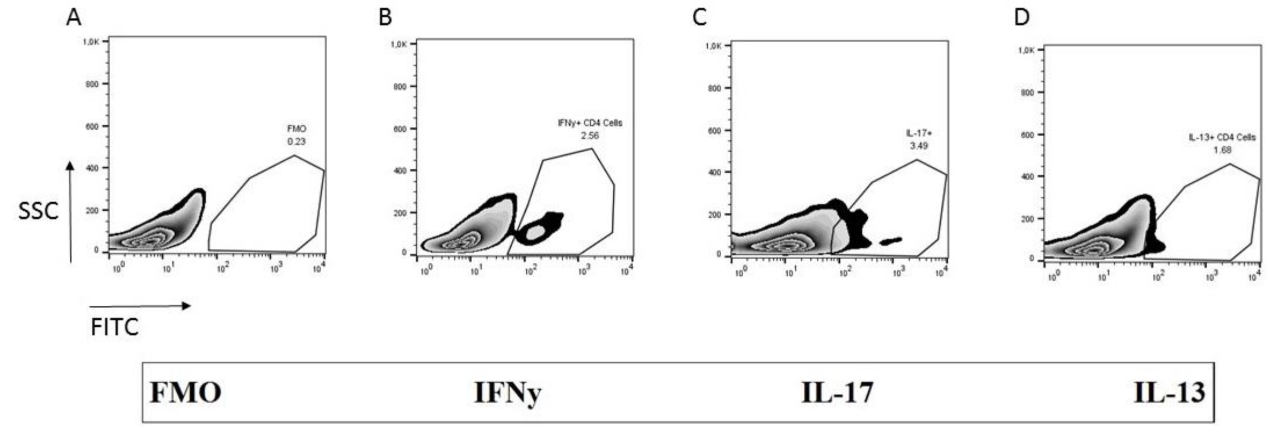

Supplementary Figure 1: Gating strategy and representative stainings for intracellular detection of Th cytokines

Mice were exposed to three cycles of DSS. Intracellular IFN-y, IL-13, IL-17A and FoxP3 expression within viable T helper cells (CD45+ CD4+) from MLN was determined by staining with mAb and flow cytometry. (A) FMO control, (B), (C) and (D) show representative staining for intracellular IFN-gamma, IL-17A and IL-13.

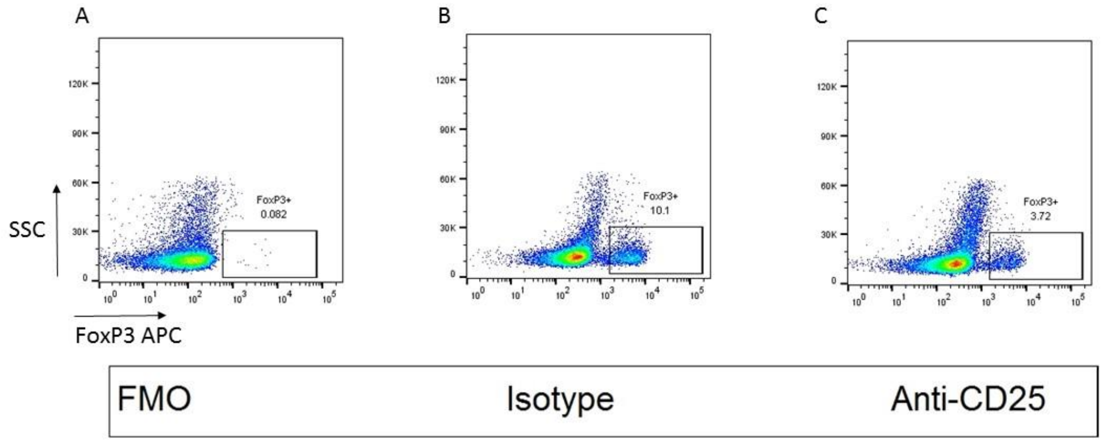

Supplementary Figure 2: Gating strategy for regulatory T cells in the lamina propria and representative example of Treg depletion by anti-CD25

Mice were exposed to three cycles of DSS to induce colitis. Regulatory T cells were depleted by anti-CD25 administration during the 2nd and 3rd cycle of DSS exposure. Lamina propria (LP) T cells were isolated from distal colon at the end of the experiment. (A) FMO on LP cells (CD45+ CD4+) from distal colon; (B)Foxp3 expression in colon LP cells from isotype treated DSS exposed mice and (C) in colon LP cells from anti-CD25 treated DSS exposed mice.

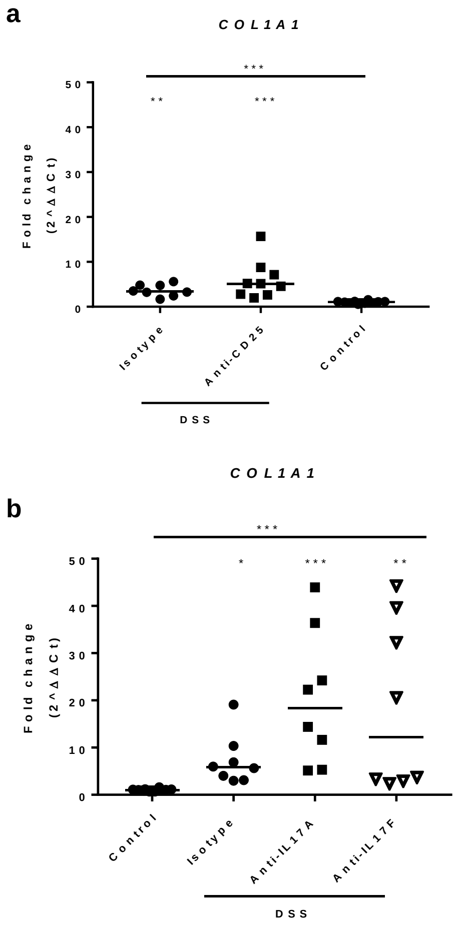

Supplementary Figure 3: Colonic gene expression of *COL1A1*

Mice were exposed to three cycles of DSS exposure and treated with anti-CD25 or anti-IL-17A of anti-IL-17F or respective isotype IgG during the second and third cycle. Control mice were not exposed to DSS. *COL1A1* colonic mRNA level are measured by RT-PCR. (A) Anti-CD25 treated DSS exposed mice (n=9), (B) anti-17A/F treated (n=9/9) DSS exposed mice, each compared to DSS exposed isotype treated mice (n=8 for each isotype) and unexposed controls (n=9) (A,B). Individual values and medians are shown. Kruskal-Wallis testing is shown over all groups. Multiple comparison with controls is shown above the group. (* p<0.05, ** p≤0.01, *** p≤0.001).

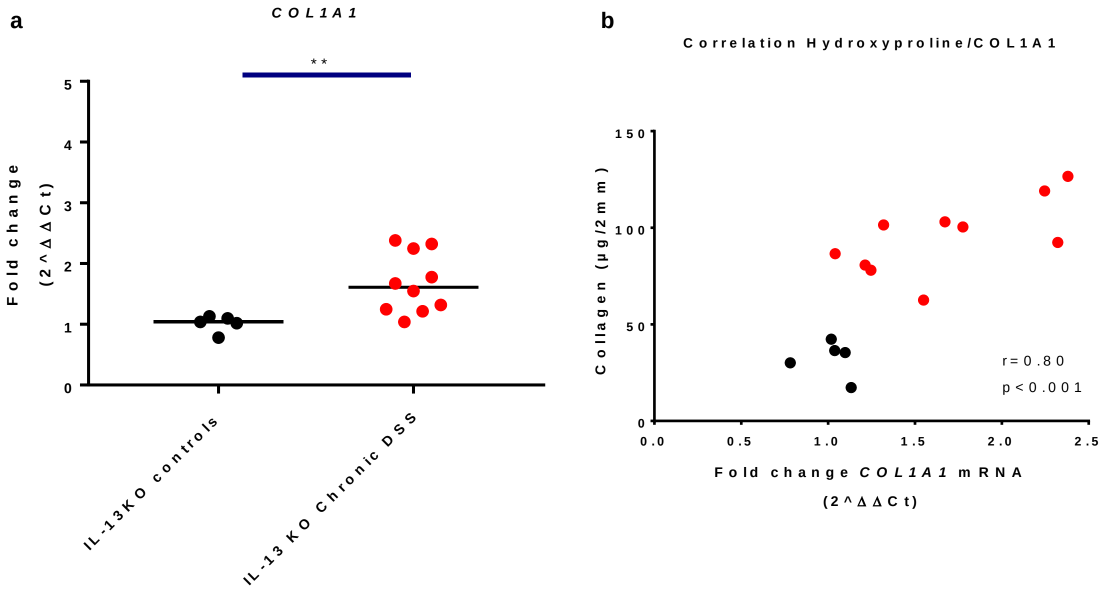

Supplementary Figure 4: Colonic gene expression of COL1A1 and its correlation with collagen protein in IL-13KO mice

Mice were exposed to three cycles of DSS exposure. (A) *COL1A1* colonic mRNA levels are measured by RT-PCR in IL-13 KO controls (n=5) as compared to IL-13 KO DSS exposed mice (n=10). (B) *COL1A1* colonic mRNA levels were correlated with colonic collagen protein levels measured by hydroxyproline assay in both IL-13KO controls (n= 5, black) and IL-13 KO DSS exposed mice (n=10, red). Individual values and medians are shown. (A) Mann-Whitney U testing (** p≤0.01). (B) Spearman rank correlation.
